# Species Distribution Models Support Distinct and Non-Random Climatic Constraints on Globally Distributed Generalist Fungi

**DOI:** 10.3390/biology15171547

**Published:** 2026-09-04

**Authors:** Mohit Mohnani, Daniel A. Henk

**Affiliations:** Centre for Evolution, Department of Life Sciences, University of Bath, Bath BA2 7AY, UK

**Keywords:** species distribution model, comparative biogeography, generalists

## Abstract

Fungi play essential roles in ecosystems as pathogens, mutualists, and ubiquitous decomposers. However, like many important microbes, the spatial distribution of species and natural populations remains poorly understood compared to plants and animals. Many fungi are described as global generalists because they occur across wide geographic areas, but it remains unclear how and if these species are constrained by climate or geographic barriers. In this study, we used Species Distribution Models to infer the global climatic suitability of three common and globally distributed fungi: *Aspergillus flavus*, *Penicillium chrysogenum* and *Aspergillus fumigatus*. Models were constructed using global occurrence data from the Global Biodiversity Information Facility and were trained with Bioclimatic variables from the WorldClim dataset. All species’ models showed high prediction fit, with predicted occurrence concentrated in the temperate and subtropical regions and broadly structured patterns. Each species showed distinct predicted distributions, but they displayed considerable spatial overlap on a global scale. Together, these results demonstrate that even apparently globally occurring and generalist fungal species occupy climatically structured niches. This study highlights the utility of SDMs and it provides a framework for future studies integrating ecological, genomics and evolutionary perspectives among the difficult to assess geographically widespread and common fungi.

## Simple Summary

When people seek to detect fungi at a location, there are some fungal species that are thought to be nearly always found, regardless of where in the world people sample. This often leads to these fungi being called generalists that grow in nearly every climate and condition. However, this has rarely been tested in a rigorous way, and it is possible that this apparent wide distribution conceals that these fungi are only able to thrive in limited environments. In this study, we use a worldwide collection database of fungi to match three of these supposed generalist fungal species to environmental conditions across the world. We use a mathematical approach, Species Distribution Modelling, to predict where in the world these fungi live and we compare between the species to determine if the predicted patterns differ between the species and if the predictions just depend on where people have collected fungi in general.

## 1. Introduction

A long-standing question in biogeography is what drives the differences between generalist and specialist species, where generalists are flexibly distributed across many different spatial environments but specialists excel in a few [1,2]. Although fungi play essential roles across terrestrial ecosystems at every spatial scale [3,4], fungal biogeography remains poorly studied compared to animals and plants [5], and assumptions persist that many fungi are generalists based on repeated observations across continents rather than quantitative assessments of environmental limits [6]. However, broad geographic distribution alone does not mean that they are environmentally flexible. Like other microbes, the apparent ubiquity in fungi may hide the underlying constraints, highlighting the need for a more quantitative approach that can evaluate the relationship between fungal occurrences and environmental conditions [7].

Species Distribution Models (SDMs) can be used to study the relationship between the distribution of multiple organisms and their environmental variables [8]. In this type of analysis, an SDM uses information about the known geographic locations of a species’ occurrences to estimate the patterns of species richness or abundance that would be expected around a defined area based on the effects of environmental factors on those patterns. SDMs are widely used in macroecological studies of plants and animals to determine their limits of distribution across the globe and to find the effects of different environmental factors on species richness or abundance [9]. However, for microbes like fungi, SDMs present several challenges compared to macroscopic animals and plants. The data used to construct SDMs for fungi may come from different sampling strategies with highly uneven sampling and inherent biases, including soil sample studies and observations from specific environments or ecosystems. In addition, true absence data is rarely if ever available for fungi [10], and fungal SDMs are based on presence-only background approaches [11].

Some specific questions about the interpretation of the results of SDMs emerge from the approaches taken. Area under the receiver operating characteristics curve (AUC) is a typical measure of accuracy for SDMs, where high values indicate good predictive power. However, high AUC may be misleading and instead indicate that there is a strong relationship between the predicted probabilities and the presence of some bias pattern or trend in measured values [12,13]. Randomised label assignments or alternate spatial configurations enable null models to more rigorously test the performance of SDM than AUC alone, and although these types of tests have already been performed on other species, this approach is not applied for fungal SDM studies despite its apparent benefits for potentially highly structured and biased sampling that can mislead AUC only approaches prone to produce good apparent model fit despite poor species discrimination and true accuracy [14]. The explicit requirement for null expected values is of particular importance when investigating taxa that are known to be generalist, as a richness of distribution may obscure underlying climatic structure [15]. With appropriate testing, comparative SDM could be used to find both shared as well as species-specific environmental associations among fungi which are considered generalists and found in broad geographical ranges.

To examine these patterns, we set out to address three main research questions: (1) Does the performance of the SDM for ‘generalist’ fungi exceed that which would be expected via null models? (2) Do widely distributed species exhibit structured and climatically constrained habitats at a global scale? (3) To what extent do SDMs of different generalist fungi reveal overlapping or distinct suitability patterns representing their shared ‘generalist’ distributions or their unique evolutionary and ecological trajectories? We selected three highly reported and well-characterized fungal species which can be found around the globe: *Aspergillus flavus*, *Aspergillus fumigatus*, and *Penicillium chrysogenum*. Their familiarity, widespread occurrence, and similarity in detectability across many natural as well as man-made habitats make them ideal organisms to compare global distribution against potentially species-specific underlying climatic constraints. To assess potential bias in modelling, we use two independent approaches, a random mixing approach and a cross-taxon approach, in which we generate a null model based on independent SDMs for phylogenetically unrelated fungi that occur at shared locations. By addressing these questions, this study aims to create a framework for investigating the climatic niches of fungi that are widely distributed.

## 2. Materials and Methods

### 2.1. Study Species

The three species of globally distributed fungi selected for this study are *Aspergillus fumigatus*, *Aspergillus flavus*, and *Penicillium chrysogenum*. These species were all selected for being frequently reported on all continents, representing a variety of ecological and functional characteristics, and being described in the literature as ecological generalists. *Aspergillus fumigatus* is a saprotrophic and opportunistic pathogenic fungus that is known to tolerate a wide range of environmental conditions [16]. *Aspergillus flavus* is widely distributed in soils and plant-associated environments, and *Penicillium chrysogenum* is a ubiquitous saprotroph of major biotechnological significance [17]. Aspergillus species have a widespread geographic distribution, making these fungi well-suited as a model for investigating whether apparent global ubiquity is associated with true climatic tolerance or is instead structured around a variety of environmental constraints.

### 2.2. Occurrence Data Collection

Occurrence data for all three species were accessed from the Global Biodiversity Information Facility (GBIF; https://www.gbif.org, accessed on 25 April 2025). Data were accessed using species-specific queries based on accepted scientific names, and occurrence tables in comma-separated values (csv) format were downloaded. Each record in the dataset includes georeferenced information (i.e., associated latitude and longitude values), as well as information on when and how the fungus was collected and associated metadata where available (https://github.com/mohnani5mohit/Fungal-Species-Distribution-Model-, accessed on 25 April 2025).

### 2.3. Occurrence Data Cleaning and Filtering

Prior to model fitting, occurrence data files were loaded and cleaned. All records that were missing georeferencing information were removed from the datasets. The values for latitude and longitude were also inspected for quality, and any records that had values outside of recognized ranges (i.e., values that would indicate that a given record may not have been accurately georeferenced) were removed from the datasets. Finally, records associated with marine environments were also removed, as only terrestrial environments were considered in these models.

To reduce the effects of spatial sampling bias and oversampling at heavily sampled locations, several data-cleaning steps were applied to the occurrence datasets prior to modelling. Duplicate records from the same geographic locations were removed to minimise the influence of repeated sampling events, and records with missing or invalid coordinates were excluded from the analysis. Records located at obvious coordinate errors, such as 0,0 coordinates or points falling outside terrestrial regions, were also removed where identified. Together, these steps helped reduce bias associated with uneven sampling intensity and improved the reliability of the species distribution models.

### 2.4. Environmental Predictor Variables

Nineteen bioclimatic variables (BIO1–BIO19) were obtained from the WorldClim v2 database at a spatial resolution of 2.5 arc-minutes (~5 km at the equator) [18,19]. These variables represent biologically meaningful summaries of temperature and precipitation patterns, including annual means, seasonality, and extreme or limiting climatic factors [20] (e.g., temperature of the coldest quarter, precipitation of the driest month).

All raster layers were resampled to a common resolution and aligned to a shared coordinate reference system prior to analysis. Marine areas were masked to restrict predictions to terrestrial environments representing our GBIF sampling strategy and fungal viability, which does not include meaningful marine data. Environmental values for each occurrence and background location were extracted using the terra package in R [21].

### 2.5. Species Distribution Modelling Framework

A Random Forest classification framework was used to construct species distribution models (SDMs) for *Aspergillus fumigatus*, *Aspergillus flavus* and *Penicillium chrysogenum*. Random Forest is an ensemble learning method that constructs multiple decision trees on different subsets of the available data [22]. This approach helps limit overfitting (i.e., fitting the model too closely to a specific dataset) and increases the robustness of predictions for other locations and conditions.

Random Forest was also selected for this project due to its previous success in completing SDM for various species and other ecological datasets [23,24]. Additionally, Random Forest models do not require the explicit identification of complex interactions between predictor variables, making it a strong candidate for modeling the impacts of environmental variables on these fungi.

### 2.6. Background Sampling and Data Preparation

The SDMs were trained using a presence–background method instead of a presence-only method (i.e., one that uses information on locations where fungi have previously been detected, as well as other randomly drawn locations across the same geographic area). The presence locations were contained within the same geographic area as the background locations.

The background areas were sampled randomly across all terrestrial systems to ensure that the models account for a variety of different environmental conditions.

To reduce the influence of any imbalances in the number of presence vs. background records used to train the models (i.e., to avoid overfitting to certain conditions if more presence vs. background records are found within certain locations), the same number of background locations were drawn as presence locations. Environmental predictor values for each record were extracted for each presence location and each background location, with each presence location assigned a label (i.e., value) of 1 and each background location assigned a label of 0 (i.e., a value indicating absence).

For each species, Random Forest models were trained on environmental predictors measured at presence and background locations. Each model used 500 trees (ntree = 500). The mtry and nodesize parameters were not manually specified and therefore used the default classification settings of the Random Forest package, corresponding to mtry = 4 for 19 predictor variables and node size = 1. Model evaluation used a 70:30 training/testing split with set.seed (123) and a classification threshold of 0.5. Background points were generated in equal numbers to presence points.

The trained models were applied to the full set of environmental rasters to generate continuous global maps of predicted environmental suitability. These predictions should be interpreted as relative suitability scores rather than probabilities of occurrence, and they represent a spatially continuous estimate of environments that are similar to those in which the species has been observed.

### 2.7. Model Evaluation and Performance Assessment

Model performance was evaluated in terms of area under the receiver operating characteristic curve (AUC). AUC is a measure of the model’s ability to distinguish between presence and background locations across all possible classification thresholds, and it is widely used in species distribution modelling. AUC values range from 0.5 (no discrimination) to 1.0 (perfect discrimination) [25].

To assess the validity of predicted SDM models, cross-validation was performed by randomly splitting available occurrence data into training and testing datasets. The models were trained on the training dataset, and their performance was evaluated on the testing dataset. This process was repeated several times to obtain reliable AUC values for model performance.

AUC values were calculated for each species, and summary statistics of model performance were computed to enable comparison of model performance across different taxonomic groups.

### 2.8. Null Model Assessment

To determine whether observed SDM model performance was due to a genuine species–environment relationship or to structural or sampling-related artefacts, two complementary null models were applied to *Aspergillus fumigatus*.

In the first null model, a label-shuffle approach was applied in which Presence–background labels were randomly permuted while retaining environmental predictors and location data unchanged. Random Forest models were fitted to these “fake” data several times, and AUC values were computed for each iteration. This process generated a null distribution of expected model performance values under a random assignment of presence/absence labels [26].

In the second null model, a cross-taxon approach was applied in which models were trained on randomly drawn occurrence data for other fungal species. The number of occurrence data points, environmental predictors, and model type used in this null model were equivalent to those used for the target species. This approach tests whether the observed AUC values for the species-of-interest are greater than those expected based on the spatial patterns exhibited by other fungal species [27].

The observed AUC values for *Aspergillus fumigatus* were compared to the two sets of null distributions to ensure that its performance exceeded that expected under the two different null models [28].

### 2.9. Multi-Species Overlap Analysis

To visualize shared and species-specific patterns of environmental suitability across the three fungal species, the predicted suitability maps for each species were combined into pairwise and multi-species overlap maps. These overlap maps show regions where multiple species exhibit high predicted suitability and can help identify climatic regions where these fungi overlap globally. Proportional land area occupancy and species overlaps were calculated from the raster results, with a presence probability over 0.5 indicating continued occupancy for each species.

### 2.10. Software and Reproducibility

All statistical analyses were performed using R (version 2024.04.2+764). Raster data processing and manipulation were performed with the terra and raster packages, while species distribution modelling was performed with the dismo package, and Random Forest models were fitted with the randomForest package. Custom R scripts were written for model evaluation and null model tests to ensure reproducibility.

## 3. Results

### 3.1. Data Coverage and Spatial Distribution

After cleaning and filtering, all three fungal species retained large, geographically extensive occurrence datasets suitable for modelling at a global scale. Removing data points with missing or impossible coordinates, marine locations, and duplicate points in the same geographic location reduced the clustering effect but preserved a broad coverage of the continents. The cleaned occurrence records for *Aspergillus fumigatus*, *Aspergillus flavus*, and *Penicillium chrysogenum* included 407, 877, and 920 occurrences respectively across multiple climatically diverse regions, such as temperate, subtropical, and tropical zones, creating a rich environment in which to train and evaluate the models. The cleaned occurrence records’ spatial distribution revealed the overlap of all three species at broad geographic scales (Europe, North America, and Asia) while also displaying species-specific hotspots and areas of abundance. This level of occurrence data allowed for the comparison of species distribution models and the evaluation of shared and distinct areas of environmental suitability.

### 3.2. Model Performance

**Figure 1:**
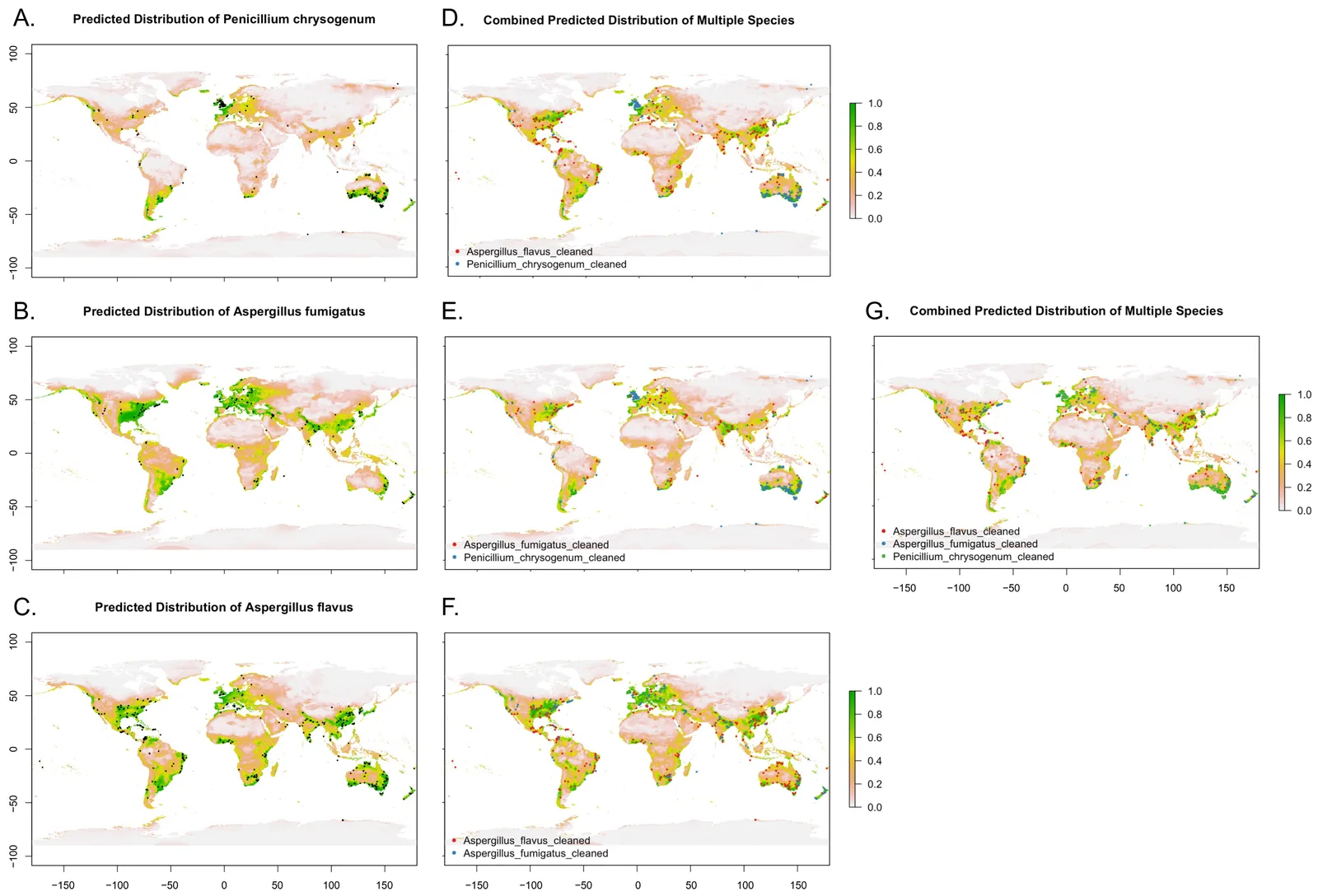
Predicted current global distributions of three fungal species using species distribution modeling (SDM) based on bioclimatic variables. (**A**) *Penicillium chrysogenum* having AUC of 0.931 and 5.5% land area occupancy. (**B**) *Aspergillus flavus* having AUC of 0.795 and 22.7% land area occupancy, and (**C**) *Aspergillus fumigatus* having AUC of 0.803 and 17.7% land area occupancy, Middle panels compare the combined distributions of two species at a time: (**D**) *Aspergillus fumigatus* and *Penicillium chrysogenum* having AUC of 0.950, 3.1% land area occupancy, and 15.4% overlap, (**E**) *Aspergillus flavus* and *Aspergillus fumigatus* having AUC of 0.83, 11.0% land area occupancy, and 37.5% overlap, and (**F**) *Aspergillus flavus* and *Penicillium chrysogenum* having AUC of 0.902, 4.6% land area occupancy, and 19.2% overlap. (**G**) shows the combined predicted distribution of all three species having AUC of 0.87, 2.87% land area occupancy, and 9.5% overlap. Color gradients represent habitat suitability probabilities ranging from 0 (unsuitable, pale pink) to 1 (highly suitable, dark green), based on Random Forest models trained with WorldClim bioclimatic variables. Points indicate known occurrence locations from GBIF.

SDMs show high performance levels across all three fungal species, with the confusion matrices for all three fungal models showing balanced classification performance, with relatively few false positives and false negatives (Table 1). The spatial models for individual and combined species were effective with the AUC values show that there is a strong presence and background discrimination ranging from 0.80 to 0.93 (Figure 1). The models show consistent performance across all training and testing datasets which suggest that there is very low to no probability of overfitting to data. The highest AUC was recorded for *Penicillium chrysogenum* compared to *Aspergillus flavus* and *Aspergillus fumigatus,* suggesting strong discrimination between presence and background under selected predictors within its distribution area.

### 3.3. Global Predicted Suitability Patterns

The predicted global suitability maps for each species revealed wide yet structured patterns of distribution, with *P. chrysogenum* covering the smallest proportion of land area (5.5%), *A. flavus* (22.7%) the most, and *A. fumigatus* (17.7%) an intermediate proportion (Figure 1A–C). In each case, the most suitable regions for the fungi were located in temperate and subtropical areas, while the unsuitable regions corresponded to areas with extreme climatic conditions, such as aridity or cold temperatures, and zones with climate-related seasonality [13,29]. However, unlike the expectation for widely distributed fungi, no species exhibited uniformly high levels of suitability across all continents. The species distribution models instead revealed regional gradients and hotspots of abundance within otherwise globally distributed species. The differences in area and configuration between the suitability maps of the different species also demonstrate that even globally distributed fungi exhibit species-specific climatic envelopes [30].

### 3.4. Pairwise and Multi-Species Overlap Patterns

The pairwise overlap maps revealed extensive overlap areas between all pairs of species, with Jaccard overlap varying between 15.4% to 37.5%, and all three species overlapping 9.5% to cover 2.87% of the land area (Figure 1D–F). The highest levels of Jaccard overlap were recorded between *Aspergillus fumigatus* and *Aspergillus flavus* across temperate regions in the Northern Hemisphere, such as Europe and North America [25]. Comparable overlaps were also recorded when comparing one of these species with *Penicillium chrysogenum*, revealing extensive shared areas for this species in temperate regions but lower levels in arid and tropical areas. The multi-species overlap map reveals that despite the shared areas across all species, there are also regions where no overlap occurs. The multi-species predicted suitability map (Figure 1G) reveals that many regions around the globe exhibit suitability for all three species, mainly restricted to temperate regions in Europe, North America, and East Asia. However, the presence of area-specific to individual species also demonstrates that these shared areas do not equate to identical ecological niches for the three species.

### 3.5. Null Model Performance and Validation

The null model tests performed on the area- and species-specific datasets provided strong supporting evidence for the observed species distribution model (SDM) performance patterns. However, they also revealed that the observed AUC values were significantly higher than those expected from alternative, random, or non-specifically structured patterns of occurrence data.

**Figure 2:**
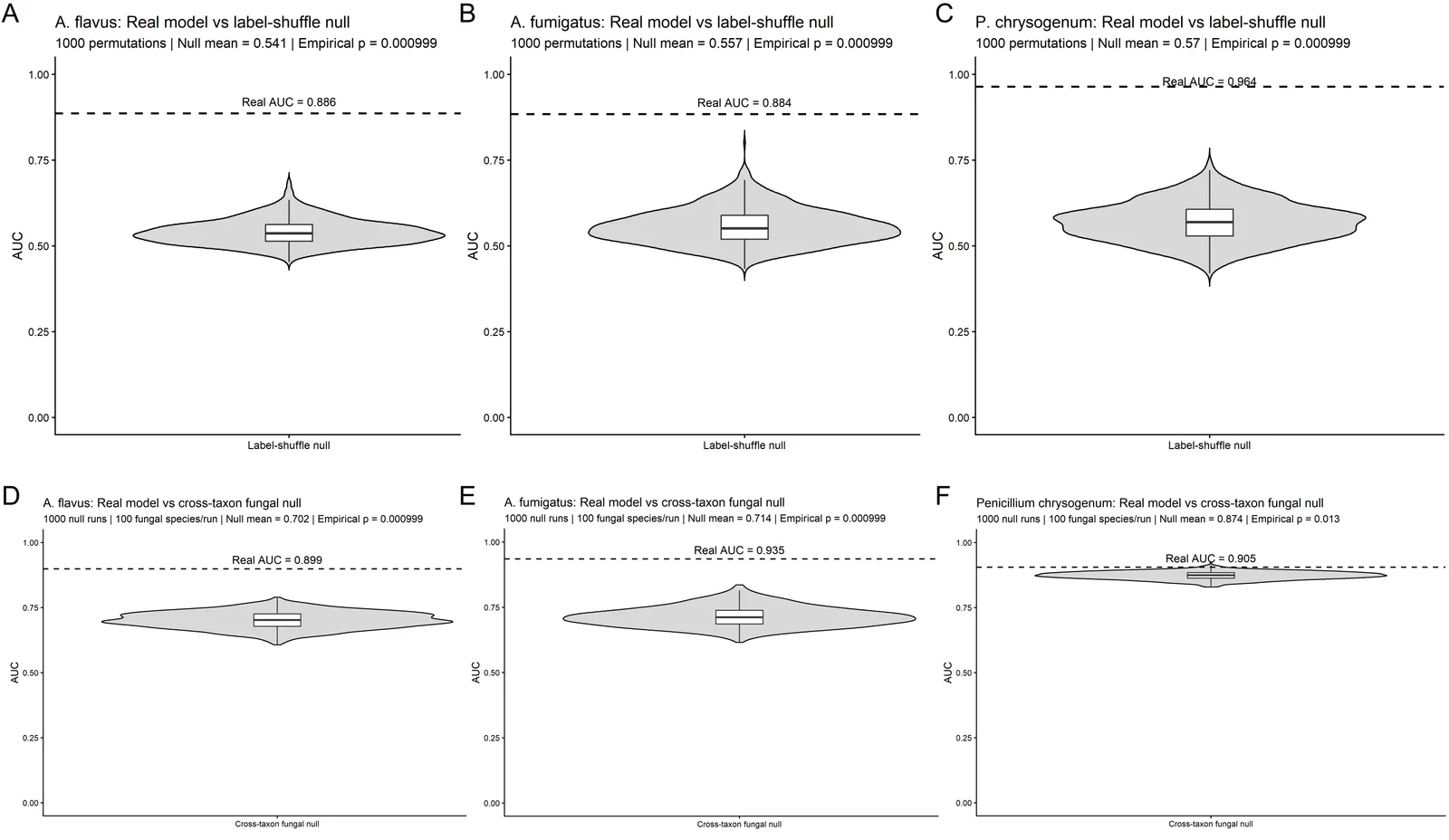
Null model evaluation for SDMs of widely distributed fungi. (**A**–**C**) Label—shuffle null test for SDM performance. The real model AUC (dashed line) exceeds all null expectations, confirming statistically meaningful predictive power. (**D**–**F**) Cross-taxon null tests for each species SDM showing real performance (dashed line) exceeds expectations based on other fungal taxa, indicating species-specific ecological structure.

In the label-shuffle null model system, for example, the AUC values measured across all iterations of shuffled label models fell into a range close to 0.5, as expected for chance-level discrimination between presence and background locations [31]. However, no replicates resulted in AUC values greater than the actual labels, resulting in *p*-values less than 0.001 compared to actual models mean differences in AUC were greater than 0.2 and standardized effect sizes greater than 5.0. A direct comparison of the observed and null AUC distributions revealed clear real vs. null model differences (Figure 2A–C), with Real AUC values above the range of the upper tail of both null models, showing that the SDM picked up species-specific ecological signal rather than effects due to spatial autocorrelation or sampling bias.

The performance of the cross-taxon null model system revealed higher AUC values compared to the label-shuffle system, indicating the background effect of fungal sampling. However, the model trained with randomly selected occurrence data from other fungal species failed to capture the specific information about the area in which the model was expected to exhibit high performance. The null model failed to achieve performance levels comparable to that of the observed SDM, producing *p*-values less than 0.001 for the more spatially dispersed samples of *Aspergillus* species and less than 0.02 for the less spatially dispersed *P. chrysogenum*. The failure of this null model system to demonstrate performance levels comparable to the focal species’ SDM model indicates that patterns or assumptions related to other fungi may not explain the observed SDM performance.

There was greater variability in AUC values across null model iterations for the cross-taxon null model than for the label-shuffle null model, showing the impact of broad-scale fungal spatial structure on model performance. However, the high performance of the best cross-taxon null models relative to the real SDM model again demonstrated the strength of the observed results.

## 4. Discussion

### 4.1. Climatic Signal Across Species

The general robustness of SDM performance across species indicated that a strong climatic signal was detected despite the differing ecological roles and geographic ranges of the species [32]. The high AUC values for all three fungi show that the climatic predictors included in the model were able to pick up important environmental constraints that limit the fungi at global scales [33]. The general consistency of model performance across species supports the general applicability of the modelling framework and suggests that similar modelling approaches may be broadly applicable across widely distributed fungi or other microbes.

### 4.2. Climatic Structure in Globally Distributed Fungi

This study shows that despite their extensive geographic ranges, the global distributions of three commonly reported fungal species—*Aspergillus fumigatus*, *Aspergillus flavus*, and *Penicillium chrysogenum*—are not independent of the environment. Rather, the findings demonstrate that these species inhabit climatically organized niches that are detectable through the use of SDMs. The SDMs showed that their anticipated flexibility is not consistent across worldwide ecosystems, despite the fact that these fungi are frequently classified as ecological generalists due to their widespread occurrence across varied habitats [34].

According to the anticipated suitability patterns, temperate and subtropical regions are where these fungi have the best environmental suitability and this is consistent with expectations based on applications of other niche modelling [35]. On the other hand, in extremely cold or extremely dry climates, applicability decreases. This trend implies that environmental limits shape the realized niches of fungi, even those with seemingly worldwide distributions. Thus, ecological neutrality or limitless environmental tolerance is not always implied by global occurrence alone. These climatic limitations make sense based on what can be inferred from species-level information such as the optimal and limiting growth temperatures from individual strains and strain collections. For example, although *A. flavus* is broadly tolerant of temperatures and water potential, it may be missing from cold, dry climates due to to the inverse relationship between required water potential and temperature and the observed near zero growth at temperatures near 2° [36]. Nevertheless, laboratory studies rarely include the breadth of samples that captures the widespread distributions of global fungi, and intraspecific variation in growth can be important, particularly near limits of growth [37], making the inverse operation of inferring climatically determined species distributions based on laboratory tests difficult.

Similar structured climatic relationships for widely scattered fungi have been discovered in earlier fungal SDM investigations [38]. These conclusions are corroborated by our findings, which also show that these patterns may be reliably identified in a variety of species with various ecological roles. The regional organization of each species’ estimated suitability varies, despite the fact that all three have exceptionally wide distributions compared to many other fungi. These variations suggest that even fungi that are frequently referred to as generalists may react to environmental gradients in diverse ways. Therefore, it is not appropriate to assume that widely spread fungi are ecologically identical, and comparative methods are useful for determining both species-specific and shared environmental drivers of fungal dispersion.

### 4.3. Importance of Rigorous Model Validation in Fungal SDMs

This study’s use of complementary null models to validate SDM performance is a significant methodological advance. Fungal occurrence records are frequently obtained through opportunistic sampling as opposed to regular surveys, which may bring spatial biases into the data. Because they capture large-scale spatial patterns rather than true ecological interactions, species distribution models may appear highly predictive in such circumstances. A crucial method for determining if observed model performance surpasses that anticipated under random or generic spatial organization is null model methods [39].

When presence–background labels were randomised, the label-shuffle null model showed that model performance decreased to almost random levels. This finding suggests that the accuracy of the relationship between occurrence locations and environmental factors determines the prediction value of the SDMs. In a similar vein, the cross-taxon null model demonstrated that models developed using occurrence data from different fungal species were unable to match the focal species’ performance. All of these findings point to species-specific ecological signals rather than general geographical patterns or sample artefacts as the cause of the observed SDM performance.

Null model techniques are still very rare in fungal SDM studies, despite their significance [33]. Without assessing whether such performance surpasses random predictions, many research mainly depend on discrimination measurements like the area under the receiver operating characteristic curve (AUC). Therefore, incorporating null model validation boosts confidence that predicted suitability patterns reflect true ecological interactions and offers a more rigorous framework for analysing SDM performance.

### 4.4. Shared and Species-Specific Environmental Suitability

Substantial overlap in anticipated appropriateness between the three fungal species was also found by the comparative SDM analysis, especially in temperate Northern Hemisphere locations. This overlap could be the result of similar environmental needs that enable these fungi to coexist in climates that are similar to one another. In fact, in temperate habitats, these species are often found together in soils, plant-associated materials, and other substrates [40].

The models did, however, also pinpoint areas where species’ suitability varied. These species-specific patterns imply that these fungi have different environmental relationships despite being widely spread [41]. Variations in ecological characteristics, substrate preferences, or physiological tolerances across species may be reflected in variations in climatic suitability patterns. The usefulness of comparative SDMs for determining common vs. lineage-specific drivers of fungal biogeography is thus highlighted by the existence of both shared and species-specific suitability patterns.

### 4.5. Limitations and Data Considerations

When evaluating these data, a number of restrictions should be taken into account. First, rather than using actual absence records, the SDMs were built using presence–background data, which could affect how the model is interpreted [42]. However, due to the scarcity of trustworthy absence data, this method is frequently employed in fungal SDM research.

Secondly, occurrence records obtained from global biodiversity databases might be dispersed unevenly throughout geographical areas and sampling initiatives [43]. Although null model testing and duplicate reduction should minimize potential biases, some residual sample bias might still exist that is not captured by these approaches, and SDMs should be viewed skeptically in the absence of additional focused sampling.

The fact that this study solely employed occurrence data from the Global Biodiversity Information Facility (GBIF) is another drawback. Attempts were made to successfully integrate occurrence data from the GlobalFungi database into the modelling workflow, but the lack of a suitable API for GlobalFungi.com limited this approach. Future research combining several fungal biodiversity databases might yield more extensive worldwide datasets.

Lastly, this study’s models only consider climate predictors, and that could mean overestimation of the climate impact when other variables may be highly correlated with climatic factors. We also did not explicitly address potential overfitting using specific approaches to reduce variable co-linearity, but the confusion matrices and null models we tested suggest that if there is overfitting, it does not impede model function to distinguish among alternatives or obscure species level differences. Nevertheless, the resolution of local-scale predictions may also be enhanced by additional environmental parameters that affect fungal distributions, such as land use, substrate availability, and biotic interactions. There are large-scale datasets that could be incorporated into combined models that allow integration of key variables, such as soil and vegetation type, allowing for detection of joint drivers of fungal distributions even at a global scale. However, SDMs are not the only way forward, with dynamic models that can incorporate more components than SDMs alone [44], and even SDMs that incorporate many environmental and biotic layers have at times been viewed skeptically, particularly for microbes [45,46,47].

### 4.6. Future Directions

By combining ecological, genetic, and functional data, future research can expand on this modelling approach and get a deeper understanding of the mechanisms controlling fungal biogeography. For instance, integrating SDMs with genomic datasets may reveal how fungal populations are shaped by environmental selection and assist in finding genetic adaptations linked to certain climatic circumstances.

These methods may also aid in the assessment of more general ecological theories, such as the long-standing microbial biogeography theory that “everything is everywhere, but the environment selects” and niche conservatism. A crucial initial step in connecting environmental distributions with evolutionary and functional mechanisms is developing strong environmental suitability models.

Thus, combining comparative SDMs with trait-based and genomic analysis is an interesting avenue for further investigation. Using the valid approaches presented here for null models, expanding the SDM approach to include individual gene distributions and their relationships to climate limitations can be scaled across species while also accounting for the sampling of available data. Uncovering how alleles, genes, or groups of genes limit apparently global microbes using these methods would enable reverse ecology to draw out the genotype to phenotype relationship in a meaningful spatial context, but these inferences must be tightly checked because they are inherently biased by sampling limitations at both genomic and spatial levels. Nonetheless, these methods can offer more profound insights into the variables that control the worldwide distribution and environmental adaptability of fungi by connecting environmental patterns with genetic and ecological processes.

## 5. Conclusions

This study demonstrates that the global biogeography of three ecologically common species of fungi is governed by structured climatic constraints rather than unconstrained environmental tolerance. Furthermore, the species distribution models exhibit high predictive performance across the three species and most importantly exceed the null expectation of both label-shuffle and cross-taxon tests. This finding confirms that SDMs can extract meaningful ecological information from fungi, even those with extremely broad geographic ranges and highly uneven sampling.

By combining the comparative SDMs with null model test validations, this study will provide a solid ecological foundation for future studies that include genetic, functional and evolutionary approaches.

## Figures and Tables

**Table 1:** Confusion matrix of fungal models.

Species	True Negative	False Positives	False Negatives	True Positives
*A. fumigatus*	45	16	8	55
*A. flavus*	100	19	35	109
*P. chrysogenum*	140	13	6	117

## Data Availability

All data and code required to reproduce the analyses presented in this study are publicly available. Species occurrence records were obtained from the Global Biodiversity Information Facility (GBIF; https://www.gbif.org). The cleaned datasets, R scripts, and analysis workflow are available at https://github.com/mohnani5mohit/Fungal-Species-Distribution-Model-, accessed on 25 April 2025.

## Abbreviations

The following abbreviations are used in this manuscript:

SDM	Species Distribution Models
API	Application Programming Interface
